# Supplementary material for: Perioperative Factors Impact on Mortality and Survival Rate of Geriatric Patients Undergoing Surgery in the COVID-19 Pandemic: A Prospective Cohort Study in Indonesia
Source: J Clin Med. 2022 Sep 8;11(18):5292. doi: 10.3390/jcm11185292 (PMC9506116; doi:10.3390/jcm11185292)
Supplement: Supplementary file 1 [file jcm-11-05292-s001.zip › jcm-1833879-supplementary.pdf]

Supplementary File S1: List of collaborators

Local collaborators: Bambang Pujo Semedi[1], Yunita Widyastuti[3], Sudadi[3], Untung Widodo[3], Akhmad Yun Jufan[3], Annisa Fadhila Farid[3], Rose Mafiana[4], Zulkifli[4], Syafruddin Gaus[6], Andi Muhammad Takdir Musba[6], Aswoco Andyk Asmoro[7], Rapto Hardian[9], Mahendra Purnama Adhi[9], Ezra Oktaliansah[10], Bastian Lubis[12],Tasrif Hamdi[12]

Data collectors: Rizky Iman Kurniawan[1], Muciento Andreas[2], Rama Josua Matasak Lolong Wulung[2], Muhammad Hanif Rahim[2], Maijoni Hendra[3], Achmad Reza Arifin[3], Feby Rianggie Dhitariza[4], Reyki Yudho Husodo[4] Nova Juwita[5], Rezki Hardiyanti[6],Nurul Huda[7], Andika Satria Praniarda[7], Achmad Hariyanto[7], Aulia Martyana Achsar[7], Dino Irawan[8], Nopian Hidayat[8], Alta Ikhsan Nur[8], Awanda Herman[8], Annisa Shaffira Pardede[8], Rohmatuah Trada Purba[9], Stefi Berlian Soefviana[10], Rossalia Yuliana[11], Agus Prima[12], M. Rizqan[13], Rizqi Ahmad Nur Dwiyono[14], Eka Satrio Putra[14]

[1] Anesthesiology and Reanimation, Faculty of Medicine, Airlangga University, Surabaya 60131, East Java, Indonesia
[2] Anesthesiology and Intensive Care, Faculty of Medicine, University of Indonesia, Jakarta 10430, Jakarta, Indonesia
[3] Anesthesiology and Intensive Therapy, Faculty of Medicine Public Health and Nursing, Gadjah Mada University, Sleman 55281, Yogyakarta, Indonesia
[4] Anesthesiology and Intensive Care, Faculty of Medicine, Sriwijaya University, Palembang 30126, South Sumatera, Indonesia
[5] Anesthesiology and Intensive Care, Faculty of Medicine, Udayana University, Denpasar 80361, Bali, Indonesia
[6] Anesthesiology, Intensive Therapy and Pain Management, Faculty of Medicine, Hasanuddin University, Makassar 90245, South Sulawesi, Indonesia
[7] Anesthesiology and Reanimation, Faculty of Medicine, Brawijaya University, Malang 65125, East Java, Indonesia
[8] Anesthesiology and Intensive Care, Faculty of Medicine, University of Riau, Pekanbaru 28133, Riau, Indonesia
[9] Anesthesiology and Intensive Care, Faculty of Medicine, Lambung Mangkurat University, Banjarmasin 70233, South Kalimantan, Indonesia
[10] Anesthesiology and Intensive Care, Faculty of Medicine, University of Padjadjaran, Bandung 45363, West Java, Indonesia
[11] Anesthesiology and Intensive Therapy, Faculty of Medicine, Syiah Kuala University, Banda Aceh 24415, Aceh, Indonesia
[12] Anesthesiology and Intensive Care, Faculty of Medicine, University of Sumatera Utara, Medan 20136, North Sumatera, Indonesia
[13] Anesthesiology and Intensive Care, Faculty of Medicine, Diponegoro University, Semarang 50275, Central Java, Indonesia
[14] Anesthesiology and Intensive Care, Faculty of Medicine, Sebelas Maret University, Surakarta 57126, Central Java, Indonesia
